# Clinical characteristics and therapeutic response of differentiated thyroid carcinoma with obesity and diabetes

**DOI:** 10.1186/s12885-023-11591-x

**Published:** 2023-11-08

**Authors:** Xuan Wang, Yang Yu, Yanhui Ji, Ziyu Ma, Jian Tan, Qiang Jia, Ning Li, Wei Zheng

**Affiliations:** Department of Nuclear Medicine, Tianjin Medical University General Hospital, Tianjin, China

**Keywords:** Differentiated thyroid carcinoma, Body mass index, Glycemic status, Obesity, Diabetes

## Abstract

**Background:**

The effects of obesity and diabetes on the clinical outcomes of differentiated thyroid cancer (DTC) remain unclear.

**Objectives:**

To explore the association between obesity and diabetes with pathological features and therapeutic response of DTC.

**Methods:**

Patients were categorized based on body mass index (BMI) and glycemic status. Compare the correlation between BMI and glycemic status with pathological features and therapeutic response of DTC. To analyze the independent risk factors for the aggressiveness of DTC.

**Results:**

The proportion of patients with bilateral tumors was higher in the overweight, obese and diabetes group (*P* = 0.001, 0.045). The overweight group demonstrated a higher TNM stage (*P* = 0.004), while the T and TNM stages were higher in the diabetes group (*P* = 0.032, 0.000). The probability of distant metastasis increases by 37.4% for each unit of BMI increase (odds ratio (OR) = 1.374, CI 95% 1.061–1.778, *P* < 0.05). The BMI of Biochemical Incomplete Response (BIR) is significantly higher than that of Excellent Response (ER) (*P* = 0.015), the fasting plasma glucose (FPG) of Structural Incomplete (SIR) was significantly higher than that of ER and BIR (*P* = 0.030, 0.014).

**Conclusion:**

Obesity and diabetes have effect on DTC aggressiveness. BMI and FPG have correlation with the therapeutic response of DTC patients.

## Background

 Differentiated thyroid cancer (DTC) is one of the most common endocrine malignancies, with its incidence rate steadily increasing over several decades [1, 2]. Most carcinomas in thyroid are well-differentiated tumors originated from follicular cells, defined as DTC, which includes papillary thyroid carcinoma (PTC) and follicular thyroid carcinoma (FTC). Around 79% of thyroid cancer cases are papillary carcinoma, and 13% are follicular carcinoma [3]. During the same period, the prevalence of obesity has risen worldwide [4]. While the increased incidence of DTC is partly attributed to heightened awareness and improved diagnostics, emerging evidence suggests that comorbidities such as obesity may also play a role [5]. Recent data indicate that, due to economic progress and improved living standards, China has become the country with the highest number of obese individuals globally, and the obesity rate continues to rise [6]. Some studies have found that obesity is independently related to the increase of DTC incidence rate [7–9], while others report no connection between the two [10, 11].

In addition, there are some studies show other risk factors for DTC, such as diabetes. Obesity is considered a promoter of type 2 diabetes mellitus (T2DM) [12], and scholars suggest that individuals with diabetes and prediabetes status should to undergo specific tumor screenings to potentially lower cancer mortality [13]. Recent research has reported strong associations between impaired fasting glucose, impaired glucose tolerance, thyroid malignancy, and poor prognosis [14, 15], while others have not found the relationship [16]. Retrospective and prospective clinical studies have produced conflicting results, and the effects of obesity and glucose metabolism on the clinical outcomes of DTC remain unclear. Body mass index (BMI) is the most widely used evaluation index for measuring obesity. Consequently, this study aimed to investigate the associations of BMI and glycemic status with pathological features and therapeutic response in DTC.

## Materials and methods

### Patients

This study evaluated a series of 1,264 consecutive DTC patients who underwent total thyroidectomy and were enrolled at the time of their first 131I treatment at Tianjin Medical University General Hospital from April 2016 to July 2020. The inclusion and exclusion criteria for this study are as follows:

Inclusion criteria:

1. Total thyroidectomy with or without lymph node dissection;
2. Pathologically confirmed DTC or lymph node metastasis from thyroid follicular cells in moderate to high-risk adult patients, all with complete pathological details;

Exclusion criteria:

1. History of neck irradiation;
2. Coexisting thyroid diseases;
3. Abnormal functions of other important organs;
4. Suffering from other tumors;
5. Previous serious mental or immune diseases.

**Fig. 1:**
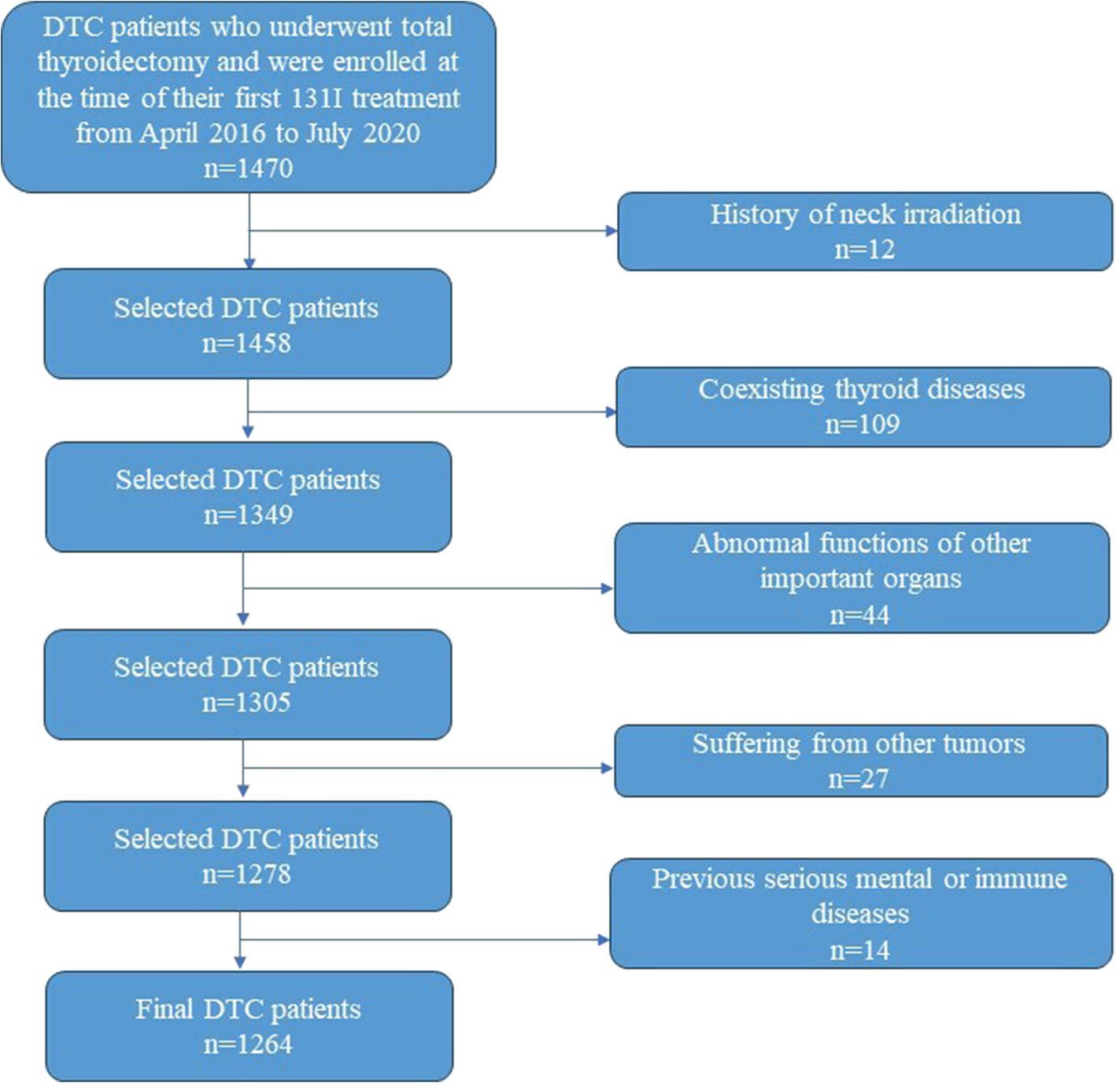
Participant flow chart

Figure 1 provides the participant flow chart. This study complies with the principles of the Declaration of Helsinki and was approved by the Ethics Committee of our hospital. All subjects participating in the study provided informed consent.

### Grouping and treatment

BMI (kg/m2) was calculated using the World Health Organization’s recommended formula: weight (kg) / height (m) squared. Determine each group based on BMI, according to the WHO recommended Chinese classification: underweight (< 18.5 kg/m^2^), normal weight (18.5–23.9 kg/m^2^), overweight (24-27.9 kg/m^2^), obese (≥ 28.0 kg/m^2^) [17]. According to the latest 8th edition of the American Joint Commission on Cancer (AJCC), all selected patients were reclassified for postoperative tumor lymph node metastasis (TNM) staging. All blood samples were taken from the subjects’ fasting peripheral venous blood in the morning for the detection of clinical indicators. The automatic chemiluminescence assay system (ARCHITECT i2000, Abbott, USA) was used to measure the serum thyroid hormone (TSH), free thyroxine (FT4), free triiodothyronine (FT3) and fasting blood glucose (FPG). Serum levels of thyroglobulin (Tg) and thyroglobulin antibody (TgAb) were assessed using an chemiluminescence immune assay system (IMMULITE_2000, Siemens). Patients were also classified into three groups based on their glycemic status, considering diabetes history and FPG values as normoglycemia (FPG < 100 mg/dL), prediabetes (FPG = 100–125 mg/dL), and diabetes (FPG ≥ 126 mg/dL) according to the ADA 2019 guideline [18]. The first ^131^I treatment was administered with an activity of 3.7 ~ 7.4 GBq of ^131^I. Patients were followed up for 16 ~ 59.6 months (37.0 ± 11.9 months on average). They were monitored at regular intervals with periodic biochemical and ultrasonographic evaluations. Based on the criteria suggested by the 2015 ATA guidelines [19], patients were divided into four groups according to their final response to therapy: Excellent Response (ER), Indeterminate Response (IR), Biochemical Incomplete Response (BIR), and Structural Incomplete Response (SIR) group.

### Statistical analysis

All statistical analyses were performed using the SPSS 26.0 software (IBM’s Statistical Product and Service Solutions). Data are expressed as the mean ± standard deviation or number (%). A t-test was applied for testing differences between means that conform to the Normal distribution. A chi-square test was used to compare differences between groups. The independent risk factors for DTC invasion were analyzed by logistic regression. A *P*-value < 0.05 was considered to indicate a statistically significant difference.

## Results

The epidemiological, clinical and pathological features are reported in Table 1. A total of 1264 DTC patients, 438 male (34.7%) and 826 female (65.3%), 1255 PTC (99.3%) and 9 FTC (0.7%), with an average age of 44.26 ± 12.51 years and a BMI of 15.8-45.8 kg/m^2^ (average 25.8 ± 4.16 kg/m^2^). Among them, 324 (25.6%) patients were obese, 495 (39.2%) patients were overweight, 420 (33.2%) patients were normal weight and 25 (2.0%) patients were underweight. Of 1264 DTC cases, 1157 (91.5%) patients were normoglycemia, 63 (5%) were prediabetes, 44 (3.5%) were diabetes.

**Table 1:** Epidemiological, clinical and pathological features of DTC patients (*n* = 1264)

Clinical characteristics	Categories	n° (%)
Sex	Male	438 (34.7%)
	Female	826 female (65.3%)
Age	Mean ± SD	44.26 ± 12.51 years
BMI(kg/m2)	underweight (< 18.5 kg/m2)	25 (2.0%)
	normal weight (18.5–23.9 kg/m2)	420 (33.2%)
	overweight (24-27.9 kg/m2)	495 (39.2%)
	obese (≥ 28.0 kg/m2)	324 (25.6%)
Glycemic Status	normoglycemia (FPG < 100 mg/dL)	1157 (91.5%)
	prediabetes (FPG = 100–125 mg/dL)	63 (5%)
	diabetes (FPG ≥ 126)	44 (3.5%)
pT	pT1	722(57.1%)
	pT2	113(8.9%)
	pT3	208(16.5%)
	pT4	221(17.5%)
pN	pN0	115(9.1%)
	pN1a	531(42.0%)
	pN1b	618(48.9%)
pM	pM0	1230(97.3%)
	pM1	34(2.7%)
Staging	I	920(72.8%)
	II	164(13.0%)
	III	104(8.2%)
	IV	76(6.0%)
Unilateral or bilateral	Unilateral	504(39.9%)
	bilateral	760(60.1%)
Therapeutic response	Excellent	271(21.4%)
	Biochemical incomplete	675(53.4%)
	Structural incomplete	284(22.5%)
	Indeterminate	34(2.7%)

*BMI *Body mass index, *FPG *Fasting plasma glucose

The analysis according to BMI evidenced overweight and obesity were more frequent in males (*P* = 0.000) while patients in the overweight group were older and had higher TNM stage (*P* = 0.000, 0.004). The proportion of patients with bilateral tumors was higher in the overweight and obese group (*P* = 0.001). However, there was no significant correlation between BMI and lymph node metastasis, TNM stage, T stage, N stage, M stage and response evaluation (*P* > 0.05) (Table 2).

**Table 2:** Clinical, laboratory and histopathologic Evaluation of DTC patients according to BMI

		BMI					
		underweight	normal weight	overweight	obese	χ^2^	P
sex	Male	0(0.0%)	84(19.2%)	188(42.9%)	166(37.9%)	94.841	0.000
	Female	25(3.0%)	336(40.7%)	307(37.2%)	158(19.1%)		
age		37.16 ± 12.91	43.57 ± 12.67	46.71 ± 12.25	41.97 ± 11.94	13.467	0.000
Unilateral or bilateral	Unilateral	14(2.8%)	192(38.1%)	300(38.7%)	221(20.4%)	17.566	0.001
	Bilateral	11(1.4%)	228(30.0%)	321(39.5%)	234(29.1%)		
lymph node metastasis	Yes	24(2.1%)	381(33.2%)	444(38.6%)	300(26.1%)	2.792	0.425
	No	1(0.9%)	39(33.9%)	51(44.3%)	24(20.9%)		
T	1	17(2.4%)	232(32.1%)	282(39.1%)	191(26.5%)	9.998	0.351
	2	1(0.9%)	38(33.6%)	43(38.1%)	31(27.4%)		
	3	6(2.9%)	81(38.9%)	77(37.0%)	44(21.2%)		
	4	1(0.5%)	69(31.2%)	93(42.1%)	58(26.2%)		
N	0	1(0.9%)	39(33.9%)	51(44.3%)	24(20.9%)	10.784	0.095
	1a	9(1.7%)	159(29.9%)	207(39.0%)	156(29.4%)		
	1b	15(2.4%)	222(35.9%)	237(38.3%)	144(23.3%)		
M	0	25(2.0%)	407(33.1%)	477(38.8%)	321(26.1%)	6.500	0.090
	1	0(0.0%)	13(38.2%)	18(52.9%)	3(8.8%)		
Staging	1	22(2.4%)	311(33.8%)	330(35.9%)	257(27.9%)	24.308	0.004
	2	1(0.6%)	49(29.9%)	79(48.2%)	35(21.3%)		
	3	0(0.0%)	32(30.8%)	49(47.1%)	23(22.1%)		
	4	2(2.6%)	28(36.8%)	37(48.7%)	9(11.8%)		
Therapeutic Response	ER	7(2.6%)	100(36.9%)	108(39.9%)	56(20.7%)	10.570	0.306
	IR	12(1.8%)	216(32.0%)	254(37.6%)	193(28.6%)		
	BIR	6(2.1%)	90(31.7%)	119(41.9%)	69(24.3%)		
	SIR	0(0.0%)	14(41.2%)	14(41.2%)	6(17.6%)		

*BMI *Body mass index, *ER *Excellent Response, *BIR *Biochemical Incomplete Response, *SIR *Structural Incomplete Response, *IR *Indeterminate Response, *DTC *Differentiated thyroid cancer

Differences in epidemiological and clinical features among the three glycemic status groups are shown in Table 3. Diabetes group are older than the other groups, and the proportion of men and bilateral tumors were found to be significantly higher (*P* = 0.000, 0.000, 0.045). Compared with the normal blood glucose group, the BMI index of patients with prediabetes and diabetes is higher, and the BMI index of patients with prediabetes is the highest (*P* = 0.000). The T stage and TNM stage were higher in the diabetes group (*P* = 0.032, 0.000). However, there was no significant correlation between glycemic status and lymph node metastasis, N stage, M stage and response evaluation (*P* > 0.05). Logistic regression analysis conducted to investigate the correlation between epidemiological or clinical features and histological features of DTC indicates that gender and age are independent risk factors for lymph node metastasis. Women are more than twice as likely to have lymph node metastasis as men and the probability of lymph node metastasis increases by 5% every year of age (odds ratio (OR) = 2.253, 1.050; CI 95% 1.366–3.717, 1.033–1.068; *P* < 0.05). BMI is an independent risk factor for distant metastasis. The probability of distant metastasis increases by 37.4% for each unit of BMI increase (odds ratio (OR) = 1.374, CI 95% 1.061–1.778, *P* < 0.05).

**Table 3:** Clinical, laboratory and histopathologic evaluation of DTC patients according to glycemic status

	GLYCEMIC STATUS					
		normoglycemia	prediabetes	diabetes	χ^2^	P
sex	Male	369(84.2%)	26(5.9%)	43(9.8%)	15.569	0.000
	Female	754(91.3%)	21(2.5%)	51(6.2%)		
age		43.28 ± 12.18	49.62 ± 13.52	53.38 ± 11.54	34.543	0.000
Unilateral or bilateral	Unilateral	461(91.5%)	16(3.2%)	27(5.4%)	6.190	0.045
	Bilateral	662(87.1%)	31(4.1%)	67(8.8%)		
lymph node metastasis	Yes	1026(89.3%)	43(3.7%)	80(7.0%)	4.125	0.127
	No	97(84.3%)	4(3.5%)	14(12.2%)		
BMI		25.55 ± 4.04	28.38 ± 5.11	27.59 ± 4.40	18.70	0.000
T	1	653(90.4%)	25(3.5%)	44(6.1%)	13.787	0.032
	2	102(90.3%)	3(2.7%)	8(7.1%)		
	3	187(89.9%)	7(3.4%)	14(6.7%)		
	4	181(81.9%)	12(5.4%)	28(12.7%)		
N	0	97(84.3%)	4(3.5%)	14(12.2%)	6.013	0.198
	1a	469(88.3%)	19(3.6%)	43(8.1%)		
	1b	557(90.1%)	24(3.9%)	37(6.0%)		
M	0	1095(89.0%)	45(3.7%)	90(7.3%)	1.486	0.476
	1	28(82.4%)	2(5.9%)	4(11.8%)		
Staging	1	850(92.4%)	27(2.9%)	43(4.7%)	49.898	0.000
	2	130(79.3%)	12(7.3%)	22(13.4%)		
	3	83(79.8%)	3(2.9%)	18(17.3%)		
	4	60(78.9%)	5(6.6%)	11(14.5%)		
Therapeutic Response	ER	243(89.7%)	12(4.4%)	16(5.9%)	7.623	0.267
	IR	608(90.1%)	22(3.3%)	45(6.7%)		
	BIR	244(85.9%)	12(4.2%)	28(9.9%)		
	SIR	28(82.4%)	1(2.9%)	5(14.7%)		

*ER *Excellent Response, *BIR *Biochemical Incomplete Response, *SIR *Structural Incomplete Response, *IR *Indeterminate Response, *DTC *Differentiated thyroid cancer

The analysis of therapeutic responses revealed significant differences in the mean values of BMI and FPG among the four groups with varying therapeutic outcomes (*P* = 0.030, *P* < 0.001). Post hoc testing results demonstrated that the BMI of the BIR group was significantly higher than that of the ER group (*P* = 0.015). Additionally, the FPG of the SIR group was notably higher than that of both the ER and BIR groups (*P* = 0.030, *P* = 0.014).

## Discussion

Obesity is a prevalent global public health issue, with its incidence steadily rising over the past two decades in both developed and developing countries [20]. A recent study by Xu et al. demonstrated that the prevalence of obesity increased from 13.4 to 35.7% between 1960 and 1962 and 2009–2010, regardless of age, sex, ethnicity, or socioeconomic status [21]. Obesity is a well-known health hazard linked to numerous malignancies [22]. Similar to obesity, DTC has exhibited a worldwide increase in incidence over the last several decades [23, 24]. Most patients with DTC have an excellent prognosis, however, 5–10% of patients have advanced disease. Patients who have aggressive and progressing metastatic DTC represent a very difficult management situation [25]. It has been suggested that DTC is associated with obesity [22], and the rise in new DTC cases in recent decades may be partially attributable to the increased prevalence of obesity [21, 26, 27]. However, association between obesity and thyroid cancer is not widely accepted.

In this retrospective analysis, we observed obesity is related to the aggressiveness of DTC. Obese patients have a higher stage and a greater probability of bilateral tumors, and with the increase of BMI, the possibility of distant metastasis will also increase. Recently, it has been reported that obesity is associated with poor pathological prognosis of several cancers [28, 29]. It cannot be ruled out that obesity will also affect the prognosis of DTC. In our study, the BMI of BIR is significantly higher than that of ER. This indicates that obese patients may have more aggressive tumors and poor prognosis. Four key factors may explain the connection between obesity and thyroid cancer: thyroid hormones, Insulin resistance, adipokines, and inflammation. Potential stimulators of TSH production, which account for the increased TSH levels in obese patients, include hormonal mediators of adipose tissue, particularly leptin [30]. As TSH is the primary stimulator of thyrocyte proliferation, this hormone could be directly involved in thyroid carcinogenesis in obese individuals [31]. The higher TSH level of obese patients will promote the tumor tissue to secrete more Tg, which will affect the patient’s condition evaluation and treatment plan decision before 131I treatment. TSH also interacts with other growth factors such as insulin. Insulin resistance is a common clinical symptom of obesity, which stimulates the production of TSH and promotes the proliferation of thyroid cancer cells [32, 33]. Adipokines or adipocytokines are a subset of cytokines produced by adipose tissue. They participate not only in immune response but also in regulating energy balance, insulin sensitivity, angiogenesis, and other processes [34, 35]. Adipose factors, such as leptin, can enhance inflammation, stimulate or block other immune molecules, and maintain the environment for tumor growth and development [36].

However, some experts believe that there is no connection between obesity and DTC [16, 37, 38]. This phenomenon might be caused by the diet, living environment, and other factors between different populations. Of course, this is also related to differences in body composition, fat distribution, and metabolic levels. In addition to the factors mentioned above, different BMI classification standards should also be considered. This study adopts the BMI classification standard suitable for the local population, and excludes the impact of different classification standards on the results.

While some studies indicate an increased risk of thyroid cancer in diabetic patients, conflicting cumulative data suggest that diabetes or prediabetes may be a risk factor for thyroid cancer [39–41]. A study shows that when DTC is combined with type 2 diabetes, the tumor is more invasive [42]. However, some studies have also obtained different results. In a study followed up for 8 years, no significant differences in clinicopathological characteristics were found between the diabetes group and the control group [43]. Our study found that the T stage and TNM stage were higher in the diabetes group. Although there is no significant relationship between glycemic status and therapeutic response, we found that the FPG of SIR was significantly higher than that of ER and BIR. Therefore, diabetes may be associated with the aggressiveness of DTC. This may be caused by a prolonged hyperglycemic state or insulin resistance. Elevated insulin levels indirectly or directly promote tumor cell proliferation and reduce apoptosis through the production of other hormones, such as insulin-like growth factor (IGF-1) [44]. Additionally, diabetes may affect the mitogenic pathway of follicular cells through the following mechanisms [39]. However, the number of diabetes in our study is relatively small, which may be related to the fact that the incidence rate of diabetes in male is higher than female [45], while the proportion of female in our research sample is higher. At the same time, it is not ruled out that some patients conceal their diabetes history.

Our study has several limitations that need to be addressed. Our study lacks information on the natural history of obesity, including BMI and glycemic status changes that occur after DTC diagnosis, as well as indicators of body composition and fat distribution. Moreover, we began data collection at the time of the first 131I treatment, which is typically performed 3–4 months after surgery. During this period, the BMI and glycemic status of our patients could be somewhat affected by a not completely adequate levothyroxine therapy. Another limitation is the small number of diabetic patients with DTC and the lack of glucose metabolism testing. These issues need further refinement in our future studies.

## Conclusion

We observed obesity and diabetes was positively associated with the aggressiveness of DTC. In addition, BMI and FPG have correlation with the therapeutic response of DTC patients. We suggest that more attention should be paid to DTC patients with obesity and diabetes. For DTC patients with obesity and diabetes, early intervention, reasonable diet, control of weight and blood sugar, and more active treatment during and after surgery should be taken. During the clinical follow-up should also control weight and blood sugar, and promote a healthy lifestyle. In the future, further evidence is needed to clarify the biological mechanism of obesity and diabetes affecting the occurrence and development of DTC.

## Data Availability

The datasets analysed during the current study are available from the corresponding author on reasonable request.
